# The Optimal Entry Point and Trajectory for Pedicle Screws to Avoid Superior Facet Joint Violation and Pedicle Penetration

**DOI:** 10.7759/cureus.72719

**Published:** 2024-10-30

**Authors:** Hüseyin Doğu

**Affiliations:** 1 Neurosurgery, Atlas University Medicine Hospital, Istanbul, TUR

**Keywords:** lumbar facet joint, lumbar spondylolisthesis, pedicle breach, pedicle screw placement, screw accuracy

## Abstract

Background

Accuracy is crucial in surgeries involving pedicle screws. This study aimed to determine the optimal screw entry points and trajectory angles for the pedicle screw technique used in the surgical treatment of lumbar instability.

Methods

To achieve this goal, a comparison was made between the screw entry points and trajectories determined using the commonly used intersection technique and those created in a three-dimensional (3D) simulation environment. Thirty-two cases of lumbar degenerative spondylolisthesis, selected from surgeries between 2018 and 2023, were included. Preoperative lumbar computed tomography (CT) images were converted into 3D models, and simulations for pedicle screw placement were conducted.

Results

Using the intersection technique, upper facet damage was noted in 31.3% of L1, 37.5% of L2, 6.3% of L3, and 31.3% of L5 segments. Adjustments to entry points and angles in the 3D environment were made to determine optimal trajectories. The revised screw angles showed statistically significant improvements at L1, L2, and L5 segments compared to the intersection technique.

Conclusions

The intersection technique does not appear safe in preserving the superior facet joint. A more lateral and caudal pedicle entry should be preferred in the upper segments, and at L5, a more lateral pedicle entry should be used. Consequently, the screw angles should be adjusted accordingly.

## Introduction

Fusion with pedicle screw technique is a common procedure in spinal surgery. This surgery has been performed using the free-hand technique up to the present day. In this technique, anatomical landmarks are crucial because the screw entry point and angle are determined based on these anatomical references [1].

One of the most critical stages of this surgery is the placement of pedicle screws in a way that avoids damaging surrounding tissues and maintains the integrity of the vertebral bone structure. To achieve this, various pedicle entry points and angles have been described [2]. Surgeries performed using these described entry points and angles can result in pedicle penetration and upper facet joint damage. In recent years, to address this issue, technological advancements have led to the use of intraoperative fluoroscopy, intraoperative computed tomography (CT), image-assisted navigation, and robotic guidance systems [3]. However, most of these systems are not widespread and are expensive. Additionally, even with these systems, it is important to determine the entry point and angle based on anatomical landmarks. Depending on the techniques and systems developed, penetration rates have been reported to be 5%-11% [3,4], while upper facet joint damage ranges from 4% to 26% [3,5]. Each of the developed techniques has its advantages and disadvantages [6].

This study aimed to determine the most appropriate pedicle entry points and screw trajectories in a virtual environment that will not cause upper facet damage or pedicle penetration in surgeries utilizing the pedicle screw technique. We aim to reveal the differences between the identified entry points and trajectories and those determined through the intersection technique.

## Materials and methods

**Figure 1:**
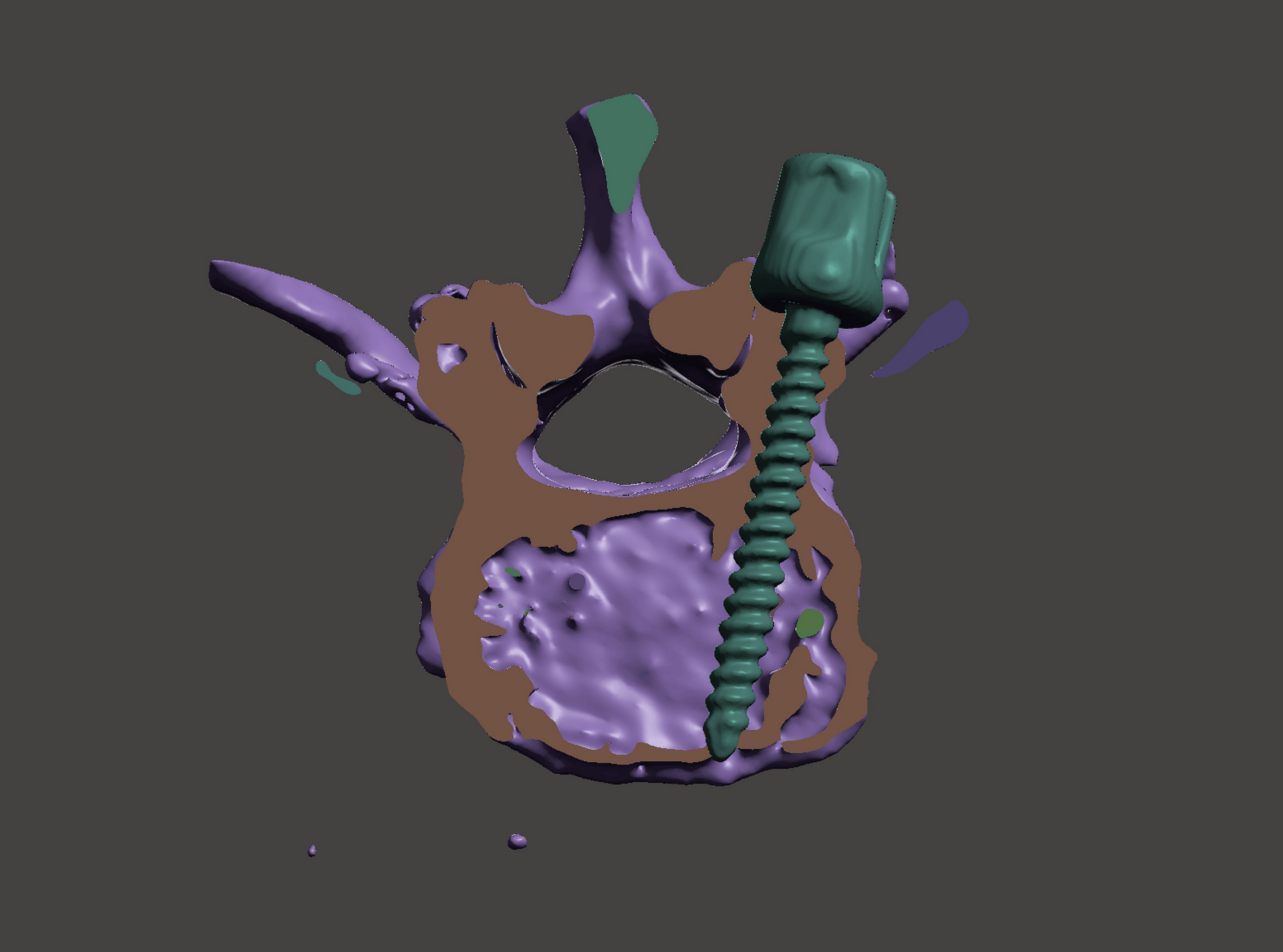
The upper facet violation caused by the pedicle screw placed using the intersection technique

**Figure 2:**
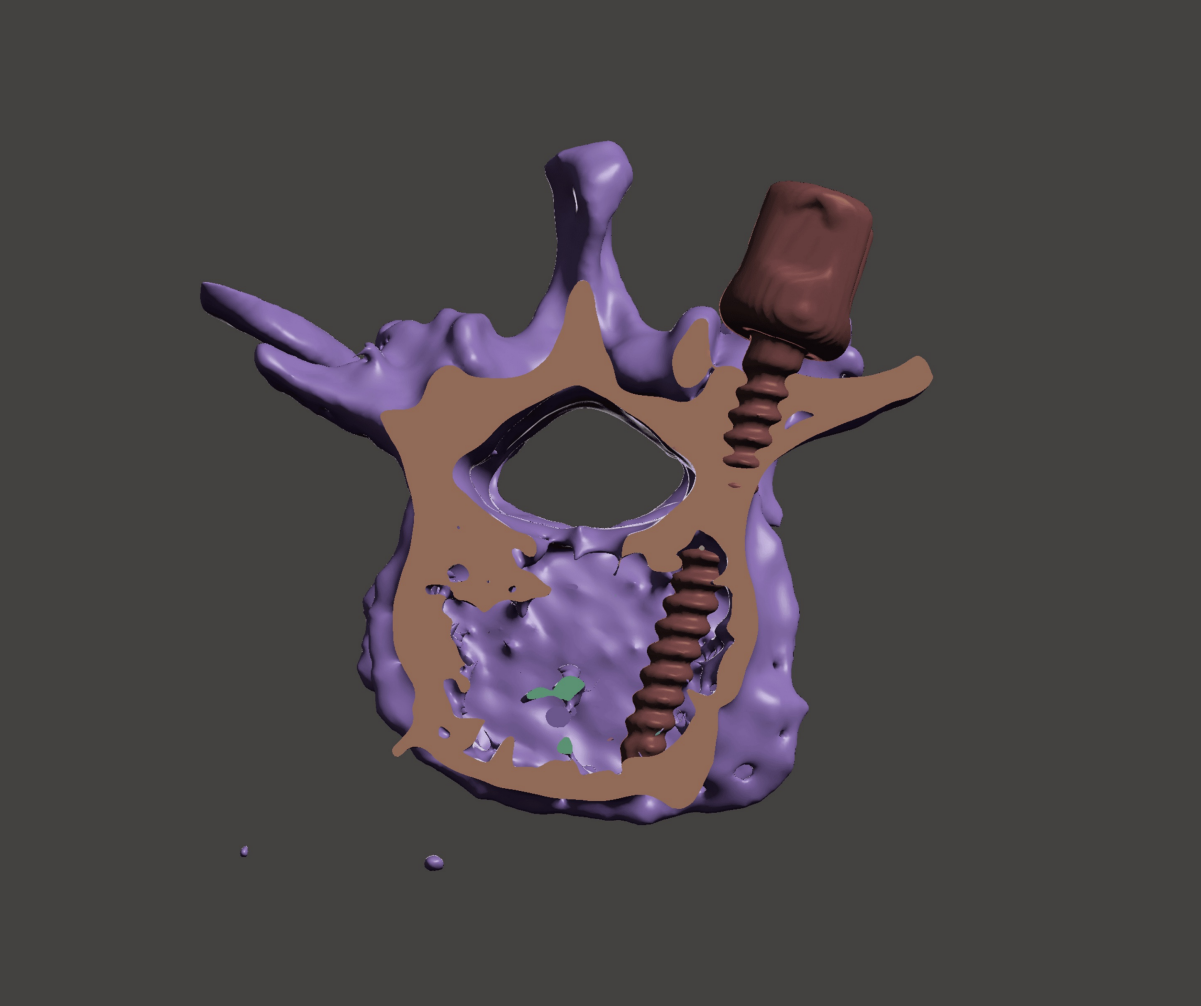
The image of the pedicle screw placed with revised entry points and angles in the 3D environment to avoid upper facet damage 3D: Three dimensional

Lumbar vertebral CT scans of 32 patients obtained from the hospital's radiological database were used in the study. To better align the cases with the study's objective, the selection was randomized among cases with degenerative listhesis operated on at our University Hospital between 2018 and 2023. Cases with advanced osteoporosis and those operated on due to revision reasons were excluded from the study. Preoperative lumbar spinal CT scans and pedicle screw data were obtained in three-dimensional (3D) file formats using 3D Slicer software (version 4.10.2). The created 3D lumbar spine and 3D fixed head pedicle screw files were transferred to another software, Meshmixer (version 3.5.474, Autodesk, Inc., San Rafael, USA), to create a suitable virtual environment for pedicle screw placement. In the simulation environment, initially, 10 pedicle screws were placed in each lumbar vertebra using the intersection technique. The intersection point of the vertical line passing through the outer edge of the facet and the horizontal line passing through the midpoint of the transverse process was used as the pedicle entry point. For L1, screws with a thickness of 5.5 mm and a length of 45 mm were used, while for the other lumbar vertebrae, screws with a thickness of 6 mm and a length of 45 mm were used. To prevent pedicle penetration, the axial sections were checked to provide the most appropriate transverse angle. The screws were placed in an optimal position, parallel to the superior end plates. The screw positions were evaluated, and the resulting upper facet joint damage was graded (Table 1) (Figure 1) [7]. In the second stage, axial sections were reviewed for segments deemed unsuitable due to upper facet joint damage to adjust the pedicle entry points, transverse angles, and, if necessary, sagittal angles to more suitable positions without adhering to any specific technique. The most ideal screw position was determined to avoid pedicle penetration and upper facet joint damage (Figure 2). Subsequently, the images from both stages were evaluated by a radiologist. The differences in pedicle entry points and transverse and sagittal angles between the intersection technique and the revised trajectory were analyzed. Statistical differences were identified. Additionally, after evaluating the patient's medical records, basic characteristics such as age and gender were also evaluated.

Statistical analysis

While evaluating the findings obtained in the study, the IBM SPSS Statistics for Windows, Version 24 (Released 2016; IBM Corp., Armonk, New York, United States) was used for statistical analysis. Furthermore, when considering the study data, quantitative variables were presented with descriptive statistics such as mean, standard deviation, median, minimum, and maximum values, while qualitative variables were presented with frequency and percentage. The normality of the data distribution was assessed using the Shapiro-Wilk test and box plot graphs. For non-normally distributed variables, the Wilcoxon signed-rank test was used to compare two follow-ups. Results were evaluated at a 95% confidence interval, and a p-value of <0.05 was accepted as statistically significant in all analyses.

**Table 1:** Upper facet joint damage grading

Grade	Description
Grade 0	The screw is not within the facet
Grade 1	The screw is in the lateral facet, but not within the facet joint
Grade 2	The screw is penetrating the facet joint
Grade 3	The screw is moving inside the facet joint

## Results

In the study, 43.8% (n = 14) of the analyzed cases were female, while 56.3% (n = 18) were male. The mean age of the cases was 65.88 ± 6.40 years (range 55-75). In a total of 32 cases with 320 screws applied using the intersection technique, upper facet joint damage occurred in 68 pedicle screws. Among these, 41 cases had grade 1 damage, 22 had grade 2 damage, and five had grade 3 damage.

**Figure 3:**
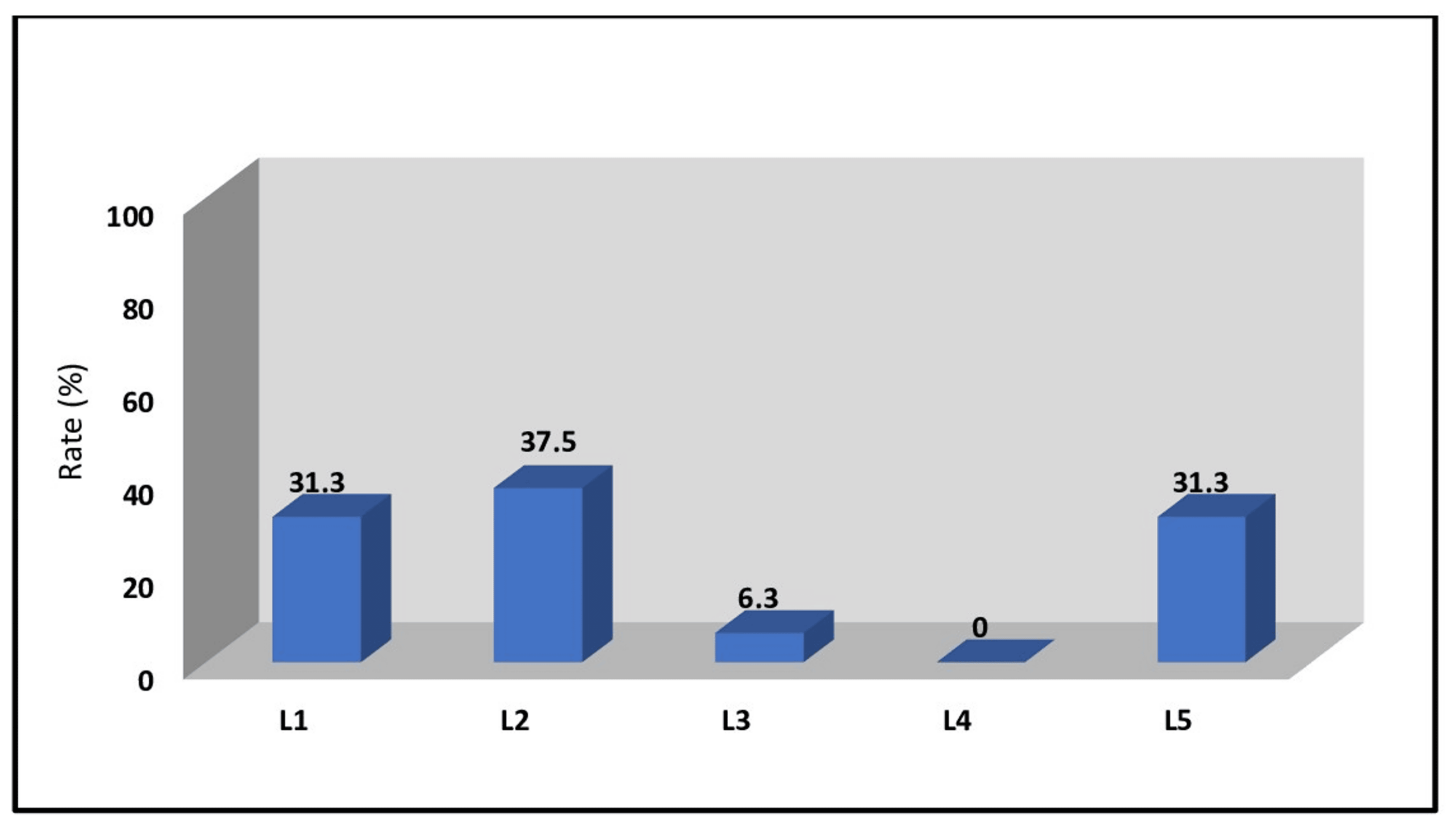
Segmental distribution of the upper facet damage caused by the application of the intersection technique L: Lumbar

Bilateral upper facet joint damage occurred in 31.3% (n = 10) of the cases at the L1 segment, 37.5% (n = 12) at the L2 segment, 6.3% (n = 2) at the L3 segment, and 31.3% (n = 10) at the L5 segment. No facet joint damage was observed at the L4 segment (Figure 3). All screws were placed without pedicle penetration using the intersection technique.

In the second stage, without adhering to any specific technique, the most suitable entry points and screw angles were determined in a 3D environment for segments with upper facet joint damage. The entry points were adjusted by shifting their positions (Table 2). Consequently, in the segments where entry points were adjusted, the screw angles were also modified, and guidance was provided with sagittal angles when necessary (Tables 3-8).

**Table 2:** Distribution of caudal and lateral shifts in pedicle entry points in all segments after revision L: Lumbar; SD: standard deviation Data are presented as mean ± SD

		L1			L2		L3		L4		L5	
		n (%)	Mean ± SD		n (%)	Mean ± SD	n (%)	Mean ± SD	n (%)	Mean ± SD	n (%)	Mean ± SD
Left caudal shift	No	22 (68.8)		20 (62.5)			30 (93.8)		32 (100.0)		32 (100.0)	
	Yes	10 (31.3)	2.10 ± 0.21	12 (37.5)		2.33 ± 0.49	2 (6.3)	2.00 ± 0.00	0 (0.0)	0.00 ± 0.00		0.00 ± 0.00
Right caudal shift	No	22 (68.8)		20 (62.5)			30 (93.8)		32 (100.0)		32 (100.0)	
	Yes	10 (31.3)	2.10 ± 0.21	12 (37.5)		2.42 ± 0.47	2 (6.3)	2.00 ± 0.00	0 (0.0)	0.00 ± 0.00		0.00 ± 0.00
Left lateral shift	No	22 (68.8)		20 (62.5)			30 (93.8)		32 (100.0)		24 (75.0)	
	Yes	10 (31.3)	2.05 ± 0.16	12 (37.5)		2.04 ± 0.14	2 (6.3)	2.00 ± 0.00	0 (0.0)	0.00 ± 0.00	8 (25.0)	2.38 ± 0.44
Right lateral shift	No	22 (68.8)		20 (62.5)			30 (93.8)		32 (100.0)		24 (75.0)	
	Yes	10 (31.3)	2.10 ± 0.21	12 (37.5)		2.25 ± 0.40	2 (6.3)	2.00 ± 0.00	0 (0.0)	0.00 ± 0.00	8 (25.0)	0.59 ± 1.07

**Table 3:** Comparison of transverse angles for pedicle screws placed using the intersection technique and revised with 3D simulation for L1 L: Lumbar; SD: standard deviation; 3D: three dimensional; min: minimum; max: maximum ^a^Wilcoxon signed-rank test, **p < 0.01 Data are presented as mean ± SD. Statistical significance was determined using a p-value threshold of p < 0.05

	Mean ± SD	Median (min-max)
L1 transverse angle left		
Intersection technique	8.36 ± 1.17	8 (7-11.5)
Revised with 3D	8.97 ± 1.62	9 (7-12.5)
Change ∆	Mean ± SD	^a^p
Revised with intersection technique-3D	0.61 ± 0.96	0.005**
L1 transverse angle right		
Intersection technique	8.42 ± 1.07	8 (7-11)
Revised with 3D	8.92 ± 1.59	9 (7-12.5)
Change ∆	Mean ± SD	^a^p
Revised with intersection technique-3D	0.50 ± 0.86	0.004**

**Table 4:** Comparison of transverse angles for pedicle screws placed using the intersection technique and revised with 3D simulation for L2 L: Lumbar; SD: standard deviation; 3D: three dimensional; min: minimum; max: maximum ^a^Wilcoxon signed-rank test, **p < 0.01 Data are presented as mean ± SD. Statistical significance was determined using a p-value threshold of p < 0.05

L2 transverse angle left		
Intersection technique	9.95 ± 1.12	10 (7-12)
Revised with 3D	10.69 ± 1.47	10 (9-14)
Change ∆	Mean ± SD	^a^p
Revised with intersection technique-3D	0.73 ± 0.97	0.001**
L2 transverse angle right		
Intersection technique	10.03 ± 1.05	10 (8-12)
Revised with 3D	10.83 ± 1.47	10.5 (9-14.5)
Change ∆	Mean ± SD	^a^p
Revised with intersection technique-3D	0.80 ± 1.04	0.001**

**Table 5:** Comparison of transverse angles for pedicle screws placed using the intersection technique and revised with 3D simulation for L3 L: Lumbar; SD: standard deviation; 3D: three dimensional; min: minimum; max: maximum ^a^Wilcoxon signed-rank test Data are presented as mean ± SD. Statistical significance was determined using a p-value threshold of p < 0.05

L3 transverse angle left		
Intersection technique	10.69 ± 0.77	11 (9.5-12)
Revised with 3D	10.84 ± 1.10	11 (9.5-14)
Change ∆	Mean ± SD	^a^p
Revised with intersection technique-3D	0.16 ± 0.77	0.276
L3 transverse angle right		
Intersection technique	10.66 ± 0.78	11 (9-12)
Revised with 3D	10.83 ± 1.05	11 (9-13.5)
Change ∆	Mean ± SD	^a^p
Revised with intersection technique-3D	0.17 ± 0.62	0.102

**Table 6:** Comparison of transverse angles for pedicle screws placed using the intersection technique and revised with 3D simulation for L4 L: Lumbar; SD: standard deviation; 3D: three dimensional; min: minimum; max: maximum ^a^Wilcoxon signed-rank test Data are presented as mean ± SD. Statistical significance was determined using a p-value threshold of p < 0.05

L4 transverse angle left		
Intersection technique	13.78 ± 1.44	13.8 (12-16.5)
Revised with 3D	13.78 ± 1.44	13.8 (12-16.5)
Change ∆	Mean ± SD	^a^p
Revised with intersection technique-3D	0.00 ± 0.00	1.000
L4 transverse angle right		
Intersection technique	13.81 ± 1.54	13.8 (12-17)
Revised with 3D	13.81 ± 1.54	13.8 (12-17)
Change ∆	Mean ± SD	^a^p
Revised with intersection technique-3D	0.00 ± 0.00	1,000

**Table 7:** Comparison of transverse angles for pedicle screws placed using the intersection technique and revised with 3D simulation for L5 L: Lumbar; SD: standard deviation; 3D: three dimensional; min: minimum; max: maximum ^a^Wilcoxon signed-rank test, **p < 0.01 Data are presented as mean ± SD. Statistical significance was determined using a p-value threshold of p < 0.05

L5 transverse angle left		
Intersection technique	23.63 ± 2.07	23.8 (20-28)
Revised with 3D	24.55 ± 1.67	24 (22.5-28)
Change ∆	Mean ± SD	^a^p
Revised with intersection technique-3D	0,92 ± 1,56	0,002**
L5 transverse angle right		
Intersection technique	23.59 ± 2.10	23.5 (20-28)
Revised with 3D	24.39 ± 2.26	24 (20-28.5)
Change ∆	Mean ± SD	^a^p
Revised with intersection technique-3D	0.80 ± 1.22	0.003**

**Table 8:** Right and left sagittal angulation values after revision L: Lumbar; SD: standard deviation Data are presented as mean ± SD

		Left	Right
L1	No; n (%)	22 (68.8)	22 (68.8)
	Yes; n (%)	10 (31.3)	10 (31.3)
	Mean ± SD	2.97 ± 0.57	3.36 ± 0.42
L2	No; n (%)	20 (62.5)	20 (6.5)
	Yes; n (%)	12 (37.5)	12 (37.5)
	Mean ± SD	4.33 ± 1.44	4.46 ± 1.27
L3	No; n (%)	30 (93.8)	30 (93.8)
	Yes; n (%)	2 (6.3)	2 (6.3)
	Mean ± SD	4.50 ± 0.71	5.00 ± 0.00
L4	No; n (%)	32 (1000)	32 (1000)
	Yes; n (%)	0 (0.0)	0 (0.0)
	Mean ± SD	0.00 ± 0.00	0.00 ± 0.00
L5	No; n (%)	32 (100.0)	32 (100.0)
	Mean ± SD	0.00 ± 0.00	0.00 ± 0.00

In the revised L1, L2, and L5 segments using the 3D simulation following the intersection technique, a statistically significant increase in transverse angles was observed for both left and right sides ((p = 0.005; p < 0.01), (p = 0.004; p < 0.01), (p = 0.001; p < 0.01), (p = 0.001; p < 0.01), (p = 0.002; p < 0.01), and (p = 0.003; p < 0.01)). The change in the left and right transverse angles in the L3 segment revised using the 3D simulation following the intersection technique was not statistically significant ((p = 0.276; p > 0.05) and (p = 0.102; p > 0.05)). Since there was no facet joint damage in the L4 segment, the screw entry point, transverse, and sagittal angles were not adjusted. In the L5 segment, the entry point was shifted laterally, and no sagittal angle adjustment was necessary.

## Discussion

Spinal fusion using pedicle screws relieves pain, protects neural structures, ensures a high fusion rate, and allows for early mobilization. However, pedicle screw fixation surgery is prone to complications due to its technical difficulties and associated risks. The most common complications are pedicle penetration and upper facet damage. These complications can negatively affect the outcome of surgical treatment. To avoid pedicle penetration and damage to the facet joint, numerous studies have been conducted, and various techniques for localizing the pedicle screw entry point have been described.

Traditionally, two intersecting reference lines have been used to determine the pedicle entry point: a vertical line from the outer edge of the facet and a horizontal line from the middle of the transverse process. In their various studies, Roy-Camille et al., Magerl, Weinstein et al., and Su et al. have described locations near the intersection of these two reference lines [8-11].

In screws placed using the traditional free-hand technique, pedicle penetration occurs at a rate of 2.6%-48.6% [12], and upper facet damage occurs at a rate of 15%-55.5% [13]. In different studies with varying entry points, pedicle penetration accuracy has been reported between 93.5% and 99.1% [2]. However, upper facet damage was not evaluated in most of these studies. Chung et al. (2007) compared the mammillary body and intersection techniques in a study of 15 cadavers and reported that the intersection technique was safer in terms of upper facet damage [14]. However, even with the intersection technique and its variants, pedicle penetration and upper facet damage can still be observed at varying rates. Even in experienced hands, the occurrence of these issues suggests that the entry points used in these techniques may not be sufficient, as the same technique is applied to all lumbar segments. Since the vertebral anatomy varies for each segment, different applications are required. The present study demonstrated that small adjustments and subsequent angle changes for each segment can significantly improve accuracy instead of using a standard entry point. Supporting the results of this study, Archavlis et al. compared two-dimensional and 3D preoperative planning in percutaneous pedicle screw placement. They recommended 3 mm lateral and 2 mm inferior entry points in the upper lumbar segments to avoid facet damage and suggested increasing the transverse angle accordingly [15]. Conversely, the present study determined the extent of lateral and caudal shifting required and the corresponding sagittal and transverse angles needed. Accordingly, it was found that 2 mm lateral and 2 mm caudal shifts in the L1, L2, and L3 segments and a 1-2 mm lateral shift in the L5 segment effectively preserved the upper facet and reduced risk. In response to shifting the pedicle entry point, it was recommended to increase the transverse angle by 0.5°-0.9° in the L1, L2, and L5 segments and to apply sagittal orientation adjustments of 2.97°-5.00° degrees in the L1, L2, and L3 segments. While the intersection technique or similarly described specific entry points may be suitable for avoiding pedicle penetration, they are not particularly appropriate for preventing upper facet damage, especially in the upper lumbar segments.

In the present study, it was determined that pedicle screws could be placed across all lumbar vertebrae without causing pedicle penetration by adjusting the transverse angles of the screws using the intersection technique. While the intersection technique is highly reliable in terms of avoiding pedicle penetration, its sensitivity in preventing upper facet damage is limited. In other words, it is not safe enough to avoid both pedicle penetration and upper facet damage. The only exception to this situation may be the L4 vertebra. In all cases, screws could be placed in the L4 vertebra using the intersection technique without causing pedicle penetration or upper facet damage.

Compared to actual surgical results, the outcomes for upper facet damage support our findings [14]. In a sense, when entry points are used based on methods like the intersection technique, upper facet damage appears to be unavoidable, with rates of 31.3% in the L1 segment, 37.5% in the L2 segment, 6.3% in the L3 segment, and 31.3% in the L5 segment. This is because, even when all sections are checked in a 3D simulation environment using the intersection technique, proper placement may still not be achieved in some cases.

This study can provide significant benefits in daily neurosurgery practice. Firstly, realizing that intersection techniques and similar methods are not ideal for pedicle screw placement encourages the adoption of more flexible and innovative management styles in surgical approaches. Additionally, it has been recognized that placing pedicle screws in all lumbar segments cannot be standardized, as some segments may require different entry points and angles. Particularly, a different approach to the upper lumbar segments is emphasized. In degenerative cases, changes in anatomical reference points can create difficulties in adjusting the entry point and angle of the pedicle; thus, it is crucial for surgeons to be aware of this situation and perform thorough preoperative preparations to enhance surgical success. Detailed preoperative planning for each vertebral segment not only provides the surgeon with confidence during the operation but also prevents complications that could arise early (in terms of pedicle penetration) and late (in terms of upper facet damage), thereby preventing the overshadowing of surgical success [16].

Limitations

Fixed head, 5.5-6 mm titanium pedicle screws were used in this study. In polyaxial screws, angling the screw head away from the facet may help preserve the facet to a greater extent. A separate study can be conducted on this topic. The information obtained in this study was derived from a simulation environment, and results may vary in actual surgical settings; therefore, further studies on this topic should be conducted.

## Conclusions

Today, technological advancements are utilized to enhance accuracy. Fluoroscopy, which has been used for a long time, has been the first step. Additionally, navigation systems that are becoming increasingly popular are being used. These technologies allow for adjustments based on the specific case rather than relying solely on fixed pedicle entry points and angles, as may be inadequate in free-hand techniques. However, based on the data obtained in the present study, pedicle entry points and screw angles can be adjusted to avoid both pedicle penetration and upper facet damage, especially in centers without access to these systems.
